# Socioeconomic Status, Protective Factors, and Mental Health Problems in Transition from Adolescence to Emerging Adulthood: Results of the Longitudinal BELLA Study

**DOI:** 10.1007/s10578-023-01582-1

**Published:** 2023-08-26

**Authors:** Jenny Maurer, Ann-Katrin Meyrose, Anne Kaman, Elvira Mauz, Ulrike Ravens-Sieberer, Franziska Reiss

**Affiliations:** 1 Department of Child and Adolescent Psychiatry, Psychotherapy, and Psychosomatics, University Medical Center Hamburg-Eppendorf, Martinistr. 52, 20246 Hamburg, Germany; 2 Developmental and Educational Psychology, Helmut Schmidt University, University of the Federal Armed Forces Hamburg, Hamburg, Germany; 3 Clinical Psychology and Psychotherapy, Helmut Schmidt University, University of the Federal Armed Forces Hamburg, Hamburg, Germany; 4 Department of Epidemiology and Health Monitoring, Robert Koch Institute, Berlin, Germany

**Keywords:** Socioeconomic inequality, Socioeconomic disparities, Resources, Self-efficacy, Social support

## Abstract

Lower familial socioeconomic status (SES) is associated with more mental health problems in adolescence. The aim of this study was to identify factors that may protect adolescents from families with lower SES from developing mental health problems in emerging adulthood. Data of the population-based longitudinal BELLA study included *n* = 426 participants aged 13 to 17 years at t0 (2009–2012) and 18 to 24 years at t1 (2014–2017). Hierarchical multiple linear regressions with interaction terms were conducted, examining three selected protective factors (self-efficacy, family climate, and social support). Self-efficacy had a small protective effect for adolescents from families with lower SES for mental health problems in emerging adulthood. However, social support had a small protective effect for adolescents from families with higher SES. No moderating effect was found for family climate. Instead, better family climate in adolescents predicted fewer mental health problems in emerging adulthood with a small effect regardless the SES in adolescence. Results indicate the need for prevention measures for adolescents from families with lower SES for becoming mentally healthy emerging adults.

**Supplementary Information:**

The online version contains supplementary material available at 10.1007/s10578-023-01582-1.

## Introduction

A large number of studies show a link between socioeconomic status (SES) and mental health problems in childhood and adolescence. Studies from Germany [1–5] as well as a review of international studies, which included studies from different European countries, North America, and Australia [6] found an inverse relationship between a low SES and mental health problems in children and adolescents. Thereby, children and adolescents living in families with a low SES are two to three times more affected by mental health problems than children and adolescents living in families with a high SES [5, 7]. Associations between low SES and internalizing problems [8], e.g., depression [9–12], and depersonalization [13] as well as externalizing problems [14], e.g., antisocial behavior [15] and ADHD [16] were found. A low SES also has an impact on the severity and persistence of mental health problems [17]. In addition, permanent family income has a significant protective effect on mental health and persistent poverty is linked to an increase in the likelihood of mental health problems [18]. Since the COVID-19 pandemic, mental health problems among children and adolescents have increased significantly (about 10% points from 18% pre-pandemic to 31% [19]), which particularly effected children and adolescents from families with low SES [20, 21].

Moreover, the period of transition between adolescence and emerging adulthood appears to be a significant time in terms of mental health problems. During this period, several mental disorders mark their onset [22, 23] and increase during this time [24]. In the phase of emerging adulthood, there are several challenges to overcome (e.g., identity explorations, feeling in-between adolescence and adulthood) that are important for mental health [25]. Therefore, the focus of this study is on this transition period from adolescence into emerging adulthood.

The research framework of this study is based on the nature and processes in human resilience (e.g., [26–30]). Resilience refers to successful and positive adaptation during development in the face of difficult challenges [27] and describes a dynamic system that withstands or recovers from these significant challenges [28]. Mental health is a criteria for a successful and positive adaptation [29]. A lower familial SES can be considered a risk factor for mental health problems [29]. According to Masten and Barnes [29], when a risk factor is present, protective factors can enable successful and positive adaptation, e.g., by having a moderating effect. Protective factors are important in developing prevention and intervention measures for children and adolescents [5, 28, 31]. These factors can refer to the child, the family, and the social environment (so-called personal, familial, and social factors) [27, 31]. However, a successful and positive adaptation in development, whereby the focus is on mental health may be influenced by various factors (e.g., trauma [32], adverse childhood experiences [33], self-regulation [34], and emotion regulation [35]) [27, 29].

As far as we know, there are only a few studies, which investigated protective factors regarding mental health in children and adolescents from families with low SES. These studies identified relevant personal protective factors (e.g., self-regulation [36], self-efficacy [37], and social competence [37, 38]) and familial protective factors (e.g., positive parenting [39] and maternal warmth [40]). In addition, the results of a cross-sectional study from Germany showed that children and adolescents from families with low SES have fewer psychosocial resources available to them and suggest that these may be especially important for children and adolescents from families with low SES [41]. Further, because previous studies have found associations between mental health problems and gender [5, 23, 42], age [23, 24], and migration background [43], these factors should be considered as well.

The aim of this study is to identify protective factors for adolescents from families with lower SES regarding their mental health in emerging adulthood based on a large German longitudinal study in order to draw conclusions for prevention and intervention measures.

Three factors were chosen as potential protective factors: *self-efficacy, family climate*, and *social support.* The personal factor, *self-efficacy* is understood as the conviction that one’s own actions can have an effect [44, 45]. The familial factor, *family climate* is characterized by good relationships between family members, cohesion and mutual support, as well as active leisure time and rules that are perceived as fair by everyone [5, 46]. Finally, the social factor *social support*, refers to the support adolescents receive from their social environment [5]; this involves sympathy and help with problems and difficulties from other people [47].

The following hypotheses were tested: First, lower familial SES in adolescence is associated with increased mental health problems in emerging adulthood. Second, this association remains even when controlling for gender, age, migration background, and mental health problems in adolescence. Third, higher self-efficacy, better family climate, and more social support in adolescence is associated with fewer mental health problems during emerging adulthood. Fourth, self-efficacy, family climate, and social support in adolescence moderate the association between SES during adolescence and mental health problems in emerging adulthood.

## Materials and Methods

### Study Design

For the present study we used data from the population-based longitudinal BELLA study, which is the mental health module of the National Health Interview and Examination Survey for Children and Adolescents (KiGGS) conducted by the Robert Koch Institute (RKI, Federal Public Health Institute of Germany) [48–51]. The BELLA sample is a randomly selected subsample of the KiGGS sample. The BELLA study includes a baseline survey (2003–2006) and four subsequent waves. The study combines a cross-sectional and longitudinal design. To maintain representativeness in the cross-sectional samples and to counteract drop-out in the longitudinal sample, new participants were recruited [50, 51]. The present analyses are based on data from the BELLA study conducted between 2009 and 2012 (referred as t0) and the 5-year follow-up conducted between 2014 and 2017 (referred as t1).

At t0, data were collected by computer-assisted interviews and subsequent questionnaires. At t1, data were collected through an online survey. Approvals by the Ethics Committee of the Hamburg Medical Association and the Federal Commissioner for Data Protection and Freedom of Information were obtained. For further details on design and methods of the BELLA study see Ravens-Sieberer et al. [50] and Otto et al. [51].

### Participants

In total 3,840 participants aged 3 to 26 years participated at t0. For the present analyses participants were included if they (i) were 13 to 17 years old at t0 (*n* = 1,076), and (ii) participated at t1 and were 18 years or older (*n* = 498), and (iii) gave valid data for all variables of interest for both measurement points (*n* = 426).

### Measures

#### Sociodemographic Variables

Sociodemographic variables comprise gender, age, migration background, and socioeconomic status. Gender (0 = male, 1 = female), age (in years), and migration background (0 = non-migration background, 1 = migration background) were assessed at t0. Migration background was determined if (i) participants and at least one parent had immigrated to Germany, or (ii) both parents had immigrated, or (iii) both parents do not have the German citizenship [52].

The SES was determined by a SES index using the most commonly used indicators of SES, i.e., parental education, parental occupation, and household income [53]. All SES indicators were assessed from parents at t0 and were equally weighted in the SES index. The index ranges from 3 to 21 with higher values indicating a higher SES.

#### Mental Health Problems

Mental health problems in adolescence (t0) were assessed by self-reports using the German Version [54] of the Strength and Difficulties Questionnaire (SDQ) [55]. The SDQ includes four subscales for mental health problems (emotional problems, conduct problems, hyperactivity and peer problems) with 5 items each. The items were offered with a three-point response scale (0 = “not true” to 2 = “certainly true”). The sum of these 20 items were calculated and build the total difficulties score, which ranges from 0 to 40. A higher score indicates more severe mental health problems. The internal consistency for the SDQ in the present study was acceptable (Cronbach’s α = .75).

The emerging adults’ mental health problems (t1) were measured by self-reports using the Symptom Checklist Shortversion 9 (SCL-S-9) [56], which is a short version of the SCL-90-R [57]. Each item of the SCL-S-9 includes a five-point response scale (0 = “not at all” to 4 = “very severe”) and refers to one of the nine scales of the SCL-90-R (somatization, obsessive-compulsive, interpersonal sensitivity, depression, anxiety, hostility, phobic anxiety, paranoid ideation, and psychoticism). A total sum score was calculated, which ranges from 0 to 36. A higher score indicates more severe mental health problems [56]. The internal consistency was good in the present study (Cronbach’s α = .86).

#### Self-efficacy

The personal protective factor *self-efficacy* in adolescents (t0) was measured by self-reports using the General Self-Efficacy Scale (GSE) [58, 59]. The ten items (e.g., “I can usually handle whatever comes my way”) were offered with a four-point response scale (0 = “not true at all” to 3 = “exactly true”). A total sum score was calculated ranging from 0 to 30. A higher score indicates higher self-efficacy. The internal consistency of the GSE was good in the present study (Cronbach’s α = .87).

#### Family Climate

The familial protective factor *family climate* (t0) was assessed by self-report using nine items of the German Family Climate Scale (FCS) [46], the German adaptation of the Family Environment Scale (FES) [60]. The items (e.g., “In our family everybody cares about each other’s worries”) were answered on a four-point response scale (0 = “not true” to 3 = “exactly true”). A total sum score was calculated ranging from 0 to 27. A higher score indicates a better family climate. The internal consistency of the FCS was good in the present study (Cronbach’s α = .82).

#### Social Support

The social factor *social support* (t0) was assessed by self-reports using the Oslo-3 Social Support Scale [47]. The scale includes the quality and the quantity of social support by measuring personal support. It consists of three items (e.g., “How many people are so close to you that you can count on them when you have serious problems?”) that asked the participants about the number of supportive people they have when they have serious problems (0 = “no one” to 3 = “more than five”), the amount of sympathy and interest of other people (0 = “no sympathy and interest” to 4 = “a lot of sympathy and interest”), and how easily they get practical help from others when help is needed (0 = “very easy” to 4 = “very difficult”). A total sum score was calculated ranging from 0 to 11. A higher score indicates higher perceived social support. The internal consistency of the Oslo-3 Social Support Scale was low in the present study (Cronbach’s α = .56).

### Data Analyses

Prior to the main analyses, descriptive analyses were carried out. These were comprised of calculating absolute and relative frequencies, means, and standard deviations for all included variables. Correlations between the factor familial SES, the control variables gender, age, and migration background, the protective factors self-efficacy, family climate, and social support and the outcome mental health problems in emerging adulthood were calculated. Following Cohen [61], a small effect was interpreted at *r* = .10, a medium effect at *r* = .30, and a large effect at *r* = .50.

For the main analyses, linear regressions with a hierarchical approach were used. Bivariate and then multiple linear regression analyses were conducted to finally have one model that includes all variables of interest. First, emerging adults’ mental health (at 5-year follow-up) were predicted by socioeconomic status in adolescence (Model 1) to replicate the evidenced association of SES and mental health [1–6]. Second, gender, age, migration background, and mental health problems in adolescence were added to the model (Model 2). Third, the protective factors self-efficacy, family climate, and social support in adolescence were included (Model 3). Fourth, interaction terms (SES × self-efficacy, SES × family climate, SES × social support) were added to the final model to test the moderation effect of self-efficacy, family climate, and social support (Model 4). The metric predictors were centered using the sample’s grand mean. Regression coefficient *B*, corresponding 95% cofidence intervals (CI), standard errors *SE(B)*, standardized coefficient β and *p*-values are reported. The standardized coefficient β was interpreted according to Cohen [61] β = .10 as small, β = .30 as medium and β = .50 as large. Adjusted *R*^*2*^ was computed for variance explanation of the model and was interpreted *R*^*2*^ *=* .02 as small, *R*^*2*^ *=* .13 as medium, and *R*^*2*^ *=* .26 as large [61]. The change of *R*^*2*^ and *F*-tests were determined for the significance of change in model fit.

Scale scores were calculated according to the manuals. Unless otherwise stated in the manual, a maximum of 30% missing values per scale was tolerated for calculation.

The significance level of all analyses was determined as α = .05. All analyses were computed using IBM SPSS Statistics Version 27®.

## Results

### Descriptive Results

The final sample was comprised of *n* = 426 adolescents aged 13 to 17 years (*M* = 15.17, *SD* = 1.28) at t0 and aged 18 to 24 years (*M* = 19.98, *SD* = 1.36) at t1. More than half of the participants were female (*n* = 249; 58.5%) and *n* = 19 (4.5%) had a migration background. For a detailed overview, see Table 1.

**Table 1:** Descriptive characteristics of the study sample

	*n* (%)	*M*	*SD*	min.	max.
Gender (t0)	426				
* female*	249 (58.5)				
* male*	177 (41.5)				
Age in adolescence (t0)	426	15.17	1.28	13	17
Age in emerging adulthood (t1)	426	19.98	1.36	18	24
Migration background (t0)	426				
* migration background*	19 (4.5)				
* non-migration background*	407 (95.5)				
SES (t0)	426	13.23	3.42	6.3	21
Self-efficacy (t0)	426	20.81	4.21	2	30
Family climate (t0)	426	15.88	4.57	2	27
Social support (t0)	426	8.46	1.63	4	11
MHP in adolescence (t0)	426	9.10	4.40	0	24
MHP in emerging adulthood (t1)	426	7.51	6.05	0	30

*Note. n* = 426; t0 = first measurement point (2009–2012); t1 = 5-year follow-up (2014–2017); min. = minimum; max. = maximum; SES = socioeconomic status; MHP = mental health problems.

### Bivariate Analysis

The bivariate analyses are presented in Table 2. The bivariate analyses revealed that lower familial SES in adolescence was associated with increased mental health problems not only in adolescence, but also at 5-year follow-up in emerging adulthood. Both showed small effects (*r* = − .173, *p* ≤ .001; *r* = − .134, *p* ≤ .01).

**Table 2:** *Bivariate correlations of SES, control variables, potential moderating protective factors, and mental health problems*

		1	2	3	4	5	6	7	8	9
1	SES (t0)	-								
2	Gender (t0)	− .047	-							
3	Age in adolescence (t0)	− .093	.014	-						
4	Migration background (t0)	.010	.002	− .033	-					
5	MHP in adolescence (t0)	**− .173** ^***^	**.212** ^***^	.012	.000	-				
6	Self-efficacy (t0)	**.133** ^**^	**− .213** ^***^	− .026	.023	**− .479** ^***^	-			
7	Family climate (t0)	.088	**− .196** ^***^	**− .150** ^**^	.051	**− .341** ^***^	**.402** ^***^	-		
8	Social support (t0)	.061	− .041	.087	.047	**− .326** ^***^	**.323** ^***^	**.325** ^***^	-	
9	MHP in emerging adulthood (t1)	**− .134** ^**^	**.175** ^***^	.059	− .036	**.298** ^***^	**− .255** ^***^	**− .288** ^***^	**− .145** ^**^	-

*Note. n* = 426; t0 = first measurement point (2009–2012); t1 = 5-year follow-up (2014–2017); SES = socioeconomic status; MHP = mental health problems; ** p ≤ .01; *** p ≤ .001; significant effects in bold.

Furthermore, higher self-efficacy, better family climate, and more social support in adolescence were associated with fewer mental health problems in adolescence with medium effects (*r* = − .479, *p* ≤ .001; *r* = − .341, *p* ≤ .001; *r* = − .326, *p* ≤ .001) and fewer mental health problems at 5-year follow-up in emerging adulthood with small to almost medium effects (*r* = − .255, *p* ≤ .001; *r* = − .288, *p* ≤ .001; *r* = − .145, *p* ≤ .01).

In addition, more mental health problems in adolescence were associated with increased mental health problems in emerging adulthood with an almost medium effect (*r* = .298, *p* ≤ .001). Female gender was associated with more mental health problems in adolescence and with increased mental health problems in emerging adulthood with small effects (*r* = .212, *p* ≤ .001; *r* = .175, *p* ≤ .001). Higher SES in adolescence was associated with higher self-efficacy in adolescence with a small effect (*r* = .133, *p* ≤ .01). All three protective factors, self-efficacy, family climate, and social support in adolescence were positively associated with each other with medium effects (*r* = .402, *p* ≤ .001; *r* = .323, *p* ≤ .001; *r* = .325, *p* ≤ .001).

### Multiple Linear Regression

An overview of the results of all four models is given in Table 3. Findings by means of Model 1 (adjusted *R*^*2*^ = .02) indicated that lower SES in adolescence was associated with increased mental health problems during emerging adulthood with a small effect (β = − .13; *p* = .006).

**Table 3:** *Regression Models 1 to 4 to predict mental health problems in emerging adulthood*

	Model 1					Model 2					Model 3					Model 4				
	*B*	*SE*	β	*p*	CI of *B*	*B*	*SE*	β	*p*	CI of *B*	*B*	*SE*	β	*p*	CI of *B*	*B*	*SE*	β	*p*	CI of *B*
(constant)	7.51	0.29		< .001	[6.94; 8.08]	7.64	1.36		< .001	[4.97; 10.31]	7.59	1.34		< .001	[4.95; 10.22]	7.35	1.34		< .001	[4.73; 9.98]
SES	-0.24	0.09	**− .13**	.006	[-0.40; -0.07]	-0.14	0.08	− .08	.091	[-0.30; 0.02]	-0.13	0.08	− .07	.113	[-0.29; 0.03]	-0.14	0.08	− .08	.097	[-0.30; 0.02]
Gender						1.42	0.58	**.12**	.014	[0.29; 2.56]	1.05	0.58	.09	.070	[-0.09; 2.18]	1.24	0.58	**.10**	.034	[0.09; 2.38]
Age						0.22	0.22	.05	.327	[-0.22; 0.65]	0.10	0.22	.02	.666	[-0.34; 0.53]	0.12	0.22	.03	.571	[-0.31; 0.56]
Migration background						-1.00	1.35	− .03	.457	[-3.65; 1.65]	-0.72	1.33	− .02	.587	[-3.33; 1.89]	-0.63	1.32	− .02	.636	[-3.23; 1.97]
MHP in adolescents						0.36	0.07	**.26**	< .001	[0.23; 0.49]	0.24	0.07	**.17**	.002	[0.09; 0.38]	0.24	0.07	**.17**	.002	[0.09; 0.38]
Self-efficacy											-0.11	0.08	− .07	.176	[-0.26; 0.05]	-0.10	0.08	− .07	.195	[-0.26; 0.05]
Family climate											-0.23	0.07	**− .17**	.001	[-0.36; -0.09]	-0.24	0.07	**− .18**	.001	[-0.37; -0.10]
Social support											-0.01	0.19	− .00	.976	[-0.37; 0.36]	-0.03	0.19	− .01	.885	[-0.39; 0.34]
SES × self-efficacy																0.05	0.02	**.10**	.043	[0.00; 0.09]
SES × family climate																-0.01	0.02	− .04	.491	[-0.05; 0.02]
SES × social support																-0.11	0.05	**− .10**	.038	[-0.21; -0.01]
Adjusted *R*^*2*^	.02					.10					.13					.14				
∆*F*(df_1_, df_2_), *p*-value	∆*F*(1, 424) = 7.773, *p* = .006					∆*F*(5, 420) = 10.580, *p* < .001					∆*F*(8, 417) = 8.956, *p*. <0.001					∆*F*(11, 414) = 7.220, *p* < .001				

*Note. n* = 426; SES = socioeconomic status; MHP = mental health problems; all predictors were assessed at t0; significant effects in bold.

Findings by means of Model 2 (adjusted *R*^*2*^ = .10) indicated that the association between a lower SES in adolescence and increased mental health problems in emerging adulthood did not remain significant (β = − .08; *p* = .091) when the control variables gender, age, migration background, and mental health problems in adolescence were entered into the model. Further, a significant but small association between female gender and increased mental health problems in emerging adulthood was examined (β = .12; *p* = .014). Increased mental health problems in adolescence were associated with more mental health problems in emerging adulthood with an almost medium effect (β = .26; *p* < .001).

Findings from Model 3 (adjusted *R*^*2*^ = .13) indicated that a better family climate in adolescence was associated with fewer mental health problems in emerging adulthood with a small effect (β = − .17; *p* = .001). The association between mental health problems in adolescence and mental health problems in emerging adulthood remained statistically significant with a small effect (β = .17; *p* = .002), whereas female gender was no longer associated with mental health problems in emerging adulthood compared to Model 2.

Finally, Model 4 (adjusted *R*^*2*^ = .14), additionally included the three interaction terms of SES during adolescence with the protective factors self-efficacy, family climate, and social support in adolescence. The results revealed small interaction effects between SES and self-efficacy in adolescence on mental health problems in emerging adulthood (β = .10; *p* = .043) as well as SES of the family and social support in adolescence on mental health problems in emerging adulthood (β = − .10; *p* = .038). The interaction of SES and family climate in adolescence was not significant. The moderator analyses revealed that higher self-efficacy was a protective factor for adolescence from families with lower SES regarding their mental health in emerging adulthood. Whereas, more social support was a protective factor for adolescents from families with higher SES regarding their mental health in emerging adulthood. Associations between female gender (β = .10; *p* = .034), mental health problems in adolescence (β = .17; *p* = .002), and family climate (β = − .18; *p* = .001) with regard to mental health problems in emerging adulthood remained statistically significant with small effects.

The final Model 4 is significant (*F* (11,414) = 7.220, *p* < .001) and explains 14% of the variance, thus, a medium amount of explained variance [61].

## Discussion

Based on the research framework of human resilience concerned with promoting resilience in people at risk for problems [26–30], this study examined protective factors (i.e., self-efficacy, family climate, and social support) that promote mental health in adolescents at risk due to lower familial SES in their transition into emerging adulthood using population-based longitudinal data from Germany.

Our study points out that adolescents from families with lower SES showed more mental health problems not only in adolescence, but also in emerging adulthood. In terms of protective factors, adolescents who had a higher self-efficacy, a better family climate, and more social support showed less mental health problems in emerging adulthood. Regarding interaction effects we found that higher self-efficacy during adolescence mitigated the effect of lower SES in adolescence on mental health problems in emerging adulthood. All single effects were small. Together, all explored factors in adolescence resulted in a medium longitudinal effect on mental health problems in emerging adulthood five years later. However, the buffering effect of social support did not seem to be present in adolescents from families with lower SES, but in adolescents from families with higher SES. This is a very interesting result, as both predictors (lower SES and less social support) are individually significantly associated with increased mental health problems in emerging adulthood.

Like our results indicate, numerous studies showed that a lower familial SES is related to more mental health problems in adolescence [6] and can be related to increased mental health problems (e.g., depression [62]) in adulthood. In our analysis, we also found this association in the first step, but it did not remain significant when further predictors were added to the model. The relationship of SES and mental health problems in adulthood seems to be more complex than a direct pathway. Self-efficacy, family climate, and social support have previously been identified as protective factors for mental health in adolescence [31]. Our bivariate analyses support this and show an association between higher self-efficacy, better family climate, and more social support in adolescence and fewer mental health problems in emerging adulthood. The directional prediction of family climate remained in the multivariate analyses.

In line with the research framework of resilience, self-efficacy can be seen as a protective factor [27]. The protective effect of self-efficacy for adolescents from families with lower SES for mental health problems in emerging adulthood goes along with the results of Meilstrup et al. [37], which surveyed students between 11 and 15 years in a cross-sectional study. Further, our bivariate analysis showed higher self-efficacy in adolescents from families with higher SES, which conversely means lower self-efficacy in adolescents from families with lower SES. Similarly, descriptive analyses of the cross-sectional KiGGS study showed that for 11-17-year-olds children and adolescents from families with low SES could draw on fewer personal resources (including self-efficacy) than those from families with medium or high SES [41]. Moreover, the results suggest that the availability of personal resources is of high importance, especially in the group of low SES with regard to mental health problems [41].

Contrary to our expectations, social support appeared to have a buffering effect on mental health problems in emerging adulthood among adolescents from families with higher SES, but not among adolescents from families with lower SES. Earlier studies also showed that children and adolescents from families with low SES have fewer social resources (i.e., social support) at their disposal [41]. Apparently more social resources were important in regard to mental health [41]. Regarding our results, it can be assumed that adolescents from families with higher SES may benefit more from social support than adolescents from families with lower SES in terms of their mental health.

Furthermore, our results indicate that adolescents from families with poorer family climate, suffer from more mental health problems in emerging adulthood regardless of SES. Since family climate has a general effect regardless of the risk factor of lower SES, the factor could also be called a promotive factor [27, 29]. Guassi Moreira and Telzer [63] showed that family cohesion during the transition from adolescence to emerging adulthood was important with regard to depressive symptoms. An increase of family cohesion during this challenging time had a positive effect and thus could be a protective factor for everyone during the significant phase of transition from adolescence into emerging adulthood.

Additionally, mental health problems in emerging adulthood are often predicted by female gender. Various studies reported that females are more affected by mental health problems than males in adolescence, especially with regard to internalizing problems [23]. Previous analyses of the BELLA study support this [5, 42]. Van Droogenbroek, Spruyt and Keppens [64] for example, assumed that gender differences in mental health problems may be due to social gender roles and the expectations and beliefs based on them. In this study, no association between age in adolescence and mental health problems in emerging adulthood was found. Thus, long term it may be more important for mental health problems in emerging adulthood what a person experienced, perceived, and received at a certain age in development rather than the age itself. Further, as discussed in the following, e.g., mental health problems in adolescence. We found that mental health problems in adolescence were the strongest predictor for mental health problems in emerging adulthood. Previous longitudinal studies showed that mental health problems have a high persistence into adolescence and adulthood [51, 65]. In addition, the review by Costello et al. [24] showed that during the period of transition from adolescence to emerging adulthood, mental health problems increase. Further, systematic reviews show growing evidence that over one-third to half of all mental health problems start before the age of 14 and almost two-third to three-quarters start before the mid-20s, which impacts not only individuals but also society [66, 67]. No associations between migration background and mental health problems in adolescence nor emerging adulthood were shown. Based on a systematic review of European studies, it could have been assumed that a migration background might be a risk factor for mental health problems in childhood and adolescence, especially in Germany [43]. However, it is emphasized that other factors probably also play an important role in this context [43, 68] (e.g., moderating effect of social capital such as social support of peers [68]).

The results lead to the assumption that adolescents from families with lower SES have a higher long-term benefit from proximal protective factors (i.e., self-efficacy) than from distal protective factors (i.e., social support, family climate). It is also possible that the influence of SES during adolescence fades when the transition to emerging adulthood occurs and thus, one’s own personal SES takes on greater importance.

### Limitations & Strengths

This study includes some limitations. Drop-out analyses of the longitudinal BELLA study showed a drop-out bias in the direction of participants of families with lower SES [51]. In these analyses, only significant differences in gender were detected, comparing the adolescents who only participated at t0 and those adolescents that participated at both measurement points (t0 and t1). Thus, more females than males participated in the study again (see Supplementary Table 1).

 Moreover, data are based on an epidemiological survey, which includes the assessment of self-reported mental health problems with brief screening instruments, but no clinical diagnoses. Nevertheless, the results can provide good indications of mental health problems in large population-based samples. Even though the same instruments were not used to survey mental health problems at both measurement points, both instruments represent the same construct. In order to survey mental health problems in an age-appropriate manner, instruments for the respective age group were selected. The internal consistency of the Oslo-3 Social Support Scale was low in this study (α = 0.56). However, this low value could be due to the multidimensionality of the instrument (quantitative and qualitative) [69, 70]. Furthermore, in a study on the standardization of the scale [71], the low value was attributed by the authors to the small number of items with reference to Buehner [72]. Despite the low internal consistency, an association between social support measured with this instrument and mental health problems could be confirmed in various studies [73–75].

In the present study, the found effects were mostly small, but predictors explained 14% of the variance in the final model (Model 4) indicating an overall medium effect. The addition of further factors influencing mental health problems in emerging adulthood could presumably explain more variance. Even though this study is based on longitudinal data, no causal relations can be proven. Finally, it must be pointed out that the results of the present study can only be transferred to other samples to a limited extent. Since in this study, the theoretical background (e.g., Reiss [6]) as well as the sample of this study does not refer to adolescents from the Global South. Further, it is important to point out that cultural aspects and the development of resilience and thus also risk and protective factors should be considered together [30, 76].

The present study has several strengths. It should be emphasized that the age range of the analyses covers a significant period in a person’s life. The transition from adolescence to emerging adulthood is significant for the development of mental health problems [22–24] and therefore an important time span for health promotion and prevention. Based on the longitudinal data of a population-based sample for Germany, we are able to give indications for an important protective factor for adolescents from families with lower SES regarding their mental health in emerging adulthood. According to the research framework of human resilience, the present study increases knowledge on resilience processes through change research [28].

### Implications

In further research, other potentially important protective factors for mental health problems in emerging adulthood in adolescents from families with lower SES should be investigated. Especially personal protective factors could be of interest (e.g. self-regulation, social competence; [36, 38]). However, other factors in adolescence may also play a role in the association between a lower SES and mental health problems, such as neurobiological processes [77]. Further, stressful life events should also be considered, because children and adolescents who are exposed to stressful life events and come from families with lower SES are more likely to develop mental health problems [78]. Furthermore, the influence of lower SES in emerging adulthood should be considered as an important factor, since in adulthood, low SES is also associated with mental health problems [79].

Implications for prevention and intervention measures can be derived from the results of this study. Thereby, adolescents from families with a lower SES are at higher risk of developing mental health problems that might persist or recur not only in emerging adulthood, but also for the rest of their lives. Early prevention and intervention measures are therefore necessary. Hence, for prevention measures in children and adolescents from families with lower SES, self-efficacy should be promoted. From the very beginning, parents and caregivers can support children in the experience that their own actions can have an impact. Prevention measures should be low threshold to reach children and adolescents from families with low education, low income or precarious employment and living conditions. Further, the school context would be exceptionally suitable, as all children and adolescents can be reached here, in particular in Germany, as school attendance is compulsory [80]. In addition, it would be beneficial if prevention would start in early childhood [5]. A good family climate should be promoted for all children and adolescents. Prevention and intervention measures may be family-based or for example may address parents directly [81].

## Summary

Adolescents from families with lower SES are at higher risk for mental health problems. In the transition from adolescence to emerging adulthood, several mental health problems begin or increase. Therefore, the aim of this study was to identify protective factors that are particularly important for adolescence from families with lower SES in the transition to emerging adulthood. Data from *n* = 426 participants from the longitudinal BELLA study were used for analyses. At t0 (2009–2012) participants were aged 13 to 17 and at t1 (2014–2017) participants were aged 18 to 24. Hierarchical multiple linear regressions with interaction terms were conducted to examine the association between the SES in adolescence and mental health problems in emerging adulthood and the effect of self-efficacy, family climate, and social support as protective factors on this association. Adolescents from families with lower SES had more mental health problems in emerging adulthood. Adolescents with higher self-efficacy, better family climate, and more social support had fewer mental health problems in emerging adulthood. When all variables were considered, the associations between better family climate in adolescence and fewer mental health problems in emerging adulthood remained. Further, a protective effect of self-efficacy for mental health problems in emerging adulthood of adolescents from families with lower SES was found. However, social support seemed to have a protective effect for adolescents from families with higher SES in regard to mental health problems in emerging adulthood, but not a protective effect for adolescents from families with lower SES. The results of this study indicate the implication that especially self-efficacy in adolescents from families with lower SES should be promoted as it seems to have a long-term impact on their mental health.

## Electronic Supplementary Material

Below is the link to the electronic supplementary material.

Supplementary Material 1

## Data Availability

The datasets generated and analyzed during the current study are not publicly available, but are available from the corresponding author on reasonable request.

## References

[1] Kuntz B, Rattay P, Poethko-Müller C et al (2018) Social inequalities in health of children and adolescents in Germany. Results of the cross-sectional KiGGS Wave 2 study. J Health Monit 3:17–33. 10.17886/RKI-GBE-2018-083 35586799 10.17886/RKI-GBE-2018-083 PMC8848913

[2] Lampert T (2011) Soziale Ungleichheit und Gesundheit im Kindes- und Jugendalter [Social inequality and health in childhood and adolescence]. Pädiatrie up2date 6:117–142

[3] Lampert T, Kuntz B, KiGGS Study Group (2015) Gesund aufwachsen – Welche Bedeutung kommt dem sozialen Status zu? [Growing up healthy – what is the significance of social status?]. GBE kompakt 6:1–22

[4] Lampert T, Kurth BM (2007) Sozialer Status und Gesundheit von Kindern und Jugendlichen: Ergebnisse des Kinder- und Jugendgesundheitssurveys (KiGGS) [Socioeconomic status and health in children and adolescents – results of the german health interview and examination survey for children and adolescents (KiGGS)]. Deutsches Ärzteblatt International 104:2944–2949

[5] Klasen F, Meyrose A-K, Otto C et al (2017) Psychische Auffälligkeiten von Kindern und Jugendlichen in Deutschland: Ergebnisse der BELLA-Studie [Mental problems of children and adolescents in Germany. Results of the BELLA study]. Monatsschr Kinderheilkd 165:402–407. 10.1007/s00112-017-0270-8

[6] Reiss F (2013) Socioeconomic inequalities and mental health problems in children and adolescents: a systematic review. Soc Sci Med 90:24–31. 10.1016/j.socscimed.2013.04.026 23746605 10.1016/j.socscimed.2013.04.026

[7] Hölling H, Schlack R, Petermann F et al (2014) Psychische Auffälligkeiten und psychosoziale Beeinträchtigungen bei Kindern und Jugendlichen im Alter von 3 bis 17 Jahren in Deutschland – Prävalenz und zeitliche Trends zu 2 Erhebungszeitpunkten (2003–2006 und 2009–2012): Ergebnisse der KiGGS-Studie – Erste Folgebefragung (KiGGS Welle 1) [Psychopathological problems and psychosocial impairment in children and adolescents aged 3–17 years in the german population: prevalence and time trends at two measurement points (2003–2006 and 2009–2012). Results of the KiGGS study: first follow-up (KiGGS Wave 1)]. Bundesgesundheitsblatt Gesundheitsforschung Gesundheitsschutz 57:807–819. 10.1007/s00103-014-1979-3 24950830 10.1007/s00103-014-1979-3

[8] Philipp J, Zeiler M, Waldherr K et al (2018) Prevalence of emotional and behavioral problems and subthreshold psychiatric disorders in austrian adolescents and the need for prevention. Soc Psychiatry Psychiatr Epidemiol 53:1325–1337. 10.1007/s00127-018-1586-y 30159723 10.1007/s00127-018-1586-y PMC6267139

[9] Angelini V, Howdon DDH, Mierau JO (2019) Childhood socioeconomic status and late-adulthood mental health: results from the survey on health, ageing and retirement in Europe. J Gerontol B Psychol Sci Soc Sci 74:95–104. 10.1093/geronb/gby028 29566242 10.1093/geronb/gby028 PMC6941210

[10] Wickrama KAS, Noh S, Elder GH (2009) An investigation of family SES-based inequalities in depressive symptoms from early adolescence to emerging adulthood. Adv Life Course Res 14:147–161. 10.1016/j.alcr.2010.04.001 10.1016/j.alcr.2010.04.001 PMC3808869 24174921

[11] Wu S, Fraser MW, Chapman MV et al (2018) Exploring the relationship between welfare participation in childhood and depression in adulthood in the United States. Soc Sci Res 76:12–22. 10.1016/j.ssresearch.2018.08.009 30268273 10.1016/j.ssresearch.2018.08.009 PMC6743494

[12] Zhou Q, Fan L, Yin Z (2018) Association between family socioeconomic status and depressive symptoms among chinese adolescents: evidence from a national household survey. Psychiatry Res 259:81–88. 10.1016/j.psychres.2017.09.072 29032165 10.1016/j.psychres.2017.09.072

[13] Michal M, Duven E, Giralt S et al (2015) Prevalence and correlates of depersonalization in students aged 12–18 years in Germany. Soc Psychiatry Psychiatr Epidemiol 50:995–1003. 10.1007/s00127-014-0957-2 25201182 10.1007/s00127-014-0957-2

[14] Pryor L, Strandberg-Larsen K, Nybo Andersen A-M et al (2019) Trajectories of family poverty and children’s mental health: results from the danish National Birth Cohort. Soc Sci Med 220:371–378. 10.1016/j.socscimed.2018.10.023 30513487 10.1016/j.socscimed.2018.10.023

[15] Piotrowska PJ, Stride CB, Maughan B et al (2019) Mechanisms underlying social gradients in child and adolescent antisocial behaviour. SSM Popul Health 7:100353. 10.1016/j.ssmph.2019.100353 30788407 10.1016/j.ssmph.2019.100353 PMC6369246

[16] Russell AE, Ford T, Williams R et al (2016) The association between socioeconomic disadvantage and attention deficit/hyperactivity disorder (ADHD): a systematic review. Child Psychiatry Hum Dev 47:440–458. 10.1007/s10578-015-0578-3 26266467 10.1007/s10578-015-0578-3

[17] McLaughlin KA, Green JG, Gruber MJ et al (2010) Childhood adversities and adult psychiatric disorders in the national comorbidity survey replication II: associations with persistence of DSM-IV disorders. Arch Gen Psychiatry 67:124–132. 10.1001/archgenpsychiatry.2009.187 20124112 10.1001/archgenpsychiatry.2009.187 PMC2847359

[18] Noonan K, Burns R, Violato M (2018) Family income, maternal psychological distress and child socio-emotional behaviour: longitudinal findings from the UK Millennium Cohort Study. SSM Popul Health 4:280–290. 10.1016/j.ssmph.2018.03.002 29854912 10.1016/j.ssmph.2018.03.002 PMC5976845

[19] Ravens-Sieberer U, Erhart M, Devine J et al (2022) Child and adolescent mental health during the COVID-19 pandemic: results of the three-wave longitudinal COPSY study. J Adolesc Health 71:570–578. 10.1016/j.jadohealth.2022.06.022 35989235 10.1016/j.jadohealth.2022.06.022 PMC9386895

[20] Ravens-Sieberer U, Kaman A, Erhart M et al (2022) Impact of the COVID-19 pandemic on quality of life and mental health in children and adolescents in Germany. Eur Child Adolesc Psychiatry 31:879–889. 10.1007/s00787-021-01726-5 33492480 10.1007/s00787-021-01726-5 PMC7829493

[21] Ravens-Sieberer U, Kaman A, Erhart M et al (2021) Quality of life and mental health in children and adolescents during the first year of the COVID-19 pandemic: results of a two-wave nationwide population-based study. Eur Child Adolesc Psychiatry. 10.1007/s00787-021-01889-1 34636964 10.1007/s00787-021-01889-1 PMC8506100

[22] Kessler RC, Angermeyer M, Anthony JC et al (2007) Lifetime prevalence and age-of-onset distributions of mental disorders in the World Health Organization’s World Mental Health Survey Initiative. World Psychiatry 6:168–176 18188442 PMC2174588

[23] Merikangas KR, He J-P, Burstein M et al (2010) Lifetime prevalence of mental disorders in U.S. adolescents: results from the National Comorbidity Survey replication–adolescent supplement (NCS-A). J Am Acad Child Adolesc Psychiatry 49:980–989. 10.1016/j.jaac.2010.05.017 20855043 10.1016/j.jaac.2010.05.017 PMC2946114

[24] Costello EJ, Copeland W, Angold A (2011) Trends in psychopathology across the adolescent years: what changes when children become adolescents, and when adolescents become adults? J Child Psychol Psychiatry 52:1015–1025. 10.1111/j.1469-7610.2011.02446.x 21815892 10.1111/j.1469-7610.2011.02446.x PMC3204367

[25] Arnett JJ, Žukauskienė R, Sugimura K (2014) The new life stage of emerging adulthood at ages 18–29 years: implications for mental health. The Lancet Psychiatry 1:569–576. 10.1016/S2215-0366(14)00080-7 26361316 10.1016/S2215-0366(14)00080-7

[26] Rutter M (1987) Psychosocial resilience and protective mechanisms. Am J Orthopsychiatry 57:316–331. 10.1111/j.1939-0025.1987.tb03541.x 3303954 10.1111/j.1939-0025.1987.tb03541.x

[27] Masten AS, Cutuli JJ, Herbers JE et al (2009) Resilience in development. In: Snyder CR, Lopez SJ (eds) The handbook of positive psychology. Oxford University Press, New York, pp 117–131

[28] Masten AS (2011) Resilience in children threatened by extreme adversity: frameworks for research, practice, and translational synergy. Dev Psychopathol 23:493–506. 10.1017/S0954579411000198 23786691 10.1017/S0954579411000198

[29] Masten AS, Barnes AJ (2018) Resilience in children: developmental perspectives. Children 5:98. 10.3390/children5070098 30018217 10.3390/children5070098 PMC6069421

[30] Masten AS, Lucke CM, Nelson KM et al (2021) Resilience in development and psychopathology: multisystem perspectives. Annu Rev Clin Psychol 17:521–549. 10.1146/annurev-clinpsy-081219-120307 33534615 10.1146/annurev-clinpsy-081219-120307

[31] Wille N, Bettge S, Ravens-Sieberer U (2008) Risk and protective factors for children’s and adolescents’ mental health: results of the BELLA study. Eur Child Adolesc Psychiatry 17 Suppl 1133–147. 10.1007/s00787-008-1015-y 10.1007/s00787-008-1015-y 19132313

[32] Lewis SJ, Arseneault L, Caspi A et al (2019) The epidemiology of trauma and post-traumatic stress disorder in a representative cohort of young people in England and Wales. Lancet Psychiatry 6:247–256. 10.1016/S2215-0366(19)30031-8 30798897 10.1016/S2215-0366(19)30031-8 PMC6384243

[33] Schilling EA, Aseltine RH, Gore S (2007) Adverse childhood experiences and mental health in young adults: a longitudinal survey. BMC Public Health 7:30. 10.1186/1471-2458-7-30 17343754 10.1186/1471-2458-7-30 PMC1832182

[34] Robson DA, Allen MS, Howard SJ (2020) Self-regulation in childhood as a predictor of future outcomes: a meta-analytic review. Psychol Bull 146:324–354. 10.1037/bul0000227 31904248 10.1037/bul0000227

[35] Berking M, Wupperman P (2012) Emotion regulation and mental health: recent findings, current challenges, and future directions. Curr Opin Psychiatry 25:128–134. 10.1097/YCO.0b013e3283503669 22262030 10.1097/YCO.0b013e3283503669

[36] Palacios-Barrios EE, Hanson JL (2019) Poverty and self-regulation: connecting psychosocial processes, neurobiology, and the risk for psychopathology. Compr Psychiatry 90:52–64. 10.1016/j.comppsych.2018.12.012 30711814 10.1016/j.comppsych.2018.12.012

[37] Meilstrup C, Holstein BE, Nielsen L et al (2020) Self-efficacy and social competence reduce socioeconomic inequality in emotional symptoms among schoolchildren. Eur J Public Health 30:80–85. 10.1093/eurpub/ckz058 31329865 10.1093/eurpub/ckz058

[38] Hosokawa R, Katsura T (2017) A longitudinal study of socioeconomic status, family processes, and child adjustment from preschool until early elementary school: the role of social competence. Child Adolesc Psychiatry Ment Health 11:62. 10.1186/s13034-017-0206-z 29270216 10.1186/s13034-017-0206-z PMC5738164

[39] Logan-Greene P, Linn B, Hartinger-Saunders R et al (2019) Understanding the ecological context of mental, emotional, and behavioral health problems: a person-centered approach. J Community Psychol 47:833–855. 10.1002/jcop.22156 30656686 10.1002/jcop.22156

[40] Kirby N, Wright B, Allgar V (2020) Child mental health and resilience in the context of socioeconomic disadvantage: results from the born in Bradford cohort study. Eur Child Adolesc Psychiatry 29:467–477. 10.1007/s00787-019-01348-y 31243580 10.1007/s00787-019-01348-y PMC7103573

[41] Schmidtke C, Geene R, Hölling H et al (2021) Mental health issues in childhood and adolescence, psychosocial resources and socioeconomic status - an analysis of the KiGGS Wave 2 data. J Health Monit 6:20–33. 10.25646/8865 35146319 10.25646/8865 PMC8734116

[42] Klasen F, Petermann F, Meyrose A-K et al (2016) Verlauf psychischer Auffälligkeiten von Kindern und Jugendlichen: Ergebnisse der BELLA-Kohortenstudie [Trajectories of mental health problems in children and adolescents: results of the BELLA cohort study]. Kindh Entwickl 25:10–20. 10.1026/0942-5403/a000184

[43] Belhadj Kouider E, Koglin U, Petermann F (2014) Emotional and behavioral problems in migrant children and adolescents in Europe: a systematic review. Eur Child Adolesc Psychiatry 23:373–391. 10.1007/s00787-013-0485-8 24132833 10.1007/s00787-013-0485-8

[44] Bandura A (1997) Self-efficacy: the exercise of control. Freeman, New York

[45] Bandura A (1977) Self-efficacy: toward a unifying theory of behavioral change. Psychol Rev 84:191–215. 10.1037/0033-295X.84.2.191 847061 10.1037//0033-295x.84.2.191

[46] Schneewind K, Beckmann M, Hecht-Jackl A (1985) Familienklima-Skalen. Bericht 8.1 und 8.2. [Family Climate Scales. Reports 8.1 and 8.2], München

[47] Brevik JI, Dalgard OS (1996) The Oslo Health Profile Inventory. University of Oslo, Oslo

[48] Kurth B-M, Kamtsiuris P, Hölling H et al (2008) The challenge of comprehensively mapping children’s health in a nation-wide health survey: design of the german KiGGS-Study. BMC Public Health 8:196. 10.1186/1471-2458-8-196 18533019 10.1186/1471-2458-8-196 PMC2442072

[49] Lange M, Hoffmann R, Mauz E et al (2018) KiGGS Wave 2 longitudinal component – data collection design and developments in the numbers of participants in the KiGGS cohort. J Health Monit 3:92–107. 10.17886/RKI-GBE-2018-035 35586182 10.17886/RKI-GBE-2018-035 PMC8848915

[50] Ravens-Sieberer U, Otto C, Kriston L et al (2015) The longitudinal BELLA study: design, methods and first results on the course of mental health problems. Eur Child Adolesc Psychiatry 24:651–663. 10.1007/s00787-014-0638-4 25428179 10.1007/s00787-014-0638-4

[51] Otto C, Reiss F, Voss C et al (2021) Mental health and well-being from childhood to adulthood: design, methods and results of the 11-year follow-up of the BELLA study. Eur Child Adolesc Psychiatry 30:1559–1577. 10.1007/s00787-020-01630-4 32918625 10.1007/s00787-020-01630-4 PMC8505294

[52] Schenk L, Ellert U, Neuhauser H (2007) Kinder und Jugendliche mit Migrationshintergrund in Deutschland: Methodische Aspekte im Kinder- und Jugendgesundheitssurvey (KiGGS) [Children and adolescents in Germany with a migration background. Methodical aspects in the german health interview and examination survey for children and adolescents (KiGGS)]. Bundesgesundheitsblatt Gesundheitsforschung Gesundheitsschutz 50:590–599. 10.1007/s00103-007-0220-z 17514443 10.1007/s00103-007-0220-z

[53] Lampert T, Müters S, Stolzenberg H et al (2014) Messung des sozioökonomischen Status in der KiGGS-Studie: Erste Folgebefragung (KiGGS Welle 1) [Measurement of socioeconomic status in the KiGGS study. First follow-up (KiGGS Wave 1)]. Bundesgesundheitsblatt Gesundheitsforschung Gesundheitsschutz 57:762–770. 10.1007/s00103-014-1974-8 24950825 10.1007/s00103-014-1974-8

[54] Klasen H, Woerner W, Rothenberger A et al (2003) Die deutsche Fassung des Strengths and Difficulties Questionnaire (SDQ-Deu) – Übersicht und Bewertung erster Validierungs- und Normierungsbefunde [The german version of the Strengths and Difficulties Questionnaire (SDQ-Deu) – overview over first validation and normative studies]. Praxis der Kinderpsychologie und Kinderpsychiatrie 52:491–502 14526759

[55] Goodman R (1997) The Strengths and Difficulties Questionnaire: a research note. J Child Psychol Psychiatry 38:581–586. 10.1111/j.1469-7610.1997.tb01545.x 9255702 10.1111/j.1469-7610.1997.tb01545.x

[56] Klaghofer R, Brähler E (2001) Konstruktion und Teststatistische Prüfung einer Kurzform der SCL-90–R [Construction and test statistical evaluation of a short version of the SCL-90–R]. Z für Klinische Psychologie Psychiatrie und Psychother 49:115–124

[57] Franke GH (2002) SCL-90-R: Symptom-Checkliste von L. R. Derogatis – deutsche Version, 2., vollständig überarbeitete und neu normierte Auflage [SCL-90-R: Symptom Checklist by L. R. Derogatis - German Version, 2nd, completely revised and newly standardized ed]. Beltz Test GmbH, Göttingen

[58] Schwarzer R, Jerusalem M (1995) Generalized self-efficacy scale. In: Weinman J, Wright S, Johnston M (eds) Measures in health psychology: a user’s portfolio. Causal and control beliefs. NFER-NELSON, Windsor, pp 35–37

[59] Schwarzer R, Jerusalem M (1999) Skalen zur Erfassung von Lehrer- und Schülermerkmalen. Dokumentation der psychometrischen Verfahren im Rahmen der wissenschaftlichen Begleitung des Modellversuchs Selbstwirksame Schulen [Scales for the assessment of teacher and student characteristics. Documentation of the psychometric procedures within the framework of the scientific monitoring of the model test self-efficant Schools]. Freie Universität Berlin, Berlin

[60] Moos RH, Moos BS (2009) Family Environment Scale manual and sampler set: development, applications and research, 4th edn. Mind Garden Inc., Palo Alto, CA

[61] Cohen J (1988) Statistical power analysis for the behavioral sciences, 2nd edn. L. Erlbaum Associates, Hillsdale, N. J.

[62] Gilman SE (2002) Socioeconomic status in childhood and the lifetime risk of major depression. Int J Epidemiol 31:359–367. 10.1093/ije/31.2.359 11980797

[63] Guassi Moreira JF, Telzer EH (2015) Changes in family cohesion and links to depression during the college transition. J Adolesc 43:72–82. 10.1016/j.adolescence.2015.05.012 26058003 10.1016/j.adolescence.2015.05.012

[64] van Droogenbroeck F, Spruyt B, Keppens G (2018) Gender differences in mental health problems among adolescents and the role of social support: results from the belgian health interview surveys 2008 and 2013. BMC Psychiatry 18:6. 10.1186/s12888-018-1591-4 29320999 10.1186/s12888-018-1591-4 PMC5763832

[65] Kessler RC, Avenevoli S, Costello EJ et al (2012) Prevalence, persistence, and sociodemographic correlates of DSM-IV disorders in the National Comorbidity Survey Replication adolescent supplement. Arch Gen Psychiatry 69:372–380. 10.1001/archgenpsychiatry.2011.160 22147808 10.1001/archgenpsychiatry.2011.160 PMC3445020

[66] Kessler RC, Berglund P, Demler O et al (2005) Lifetime prevalence and age-of-onset distributions of DSM-IV disorders in the National Comorbidity Survey Replication. Arch Gen Psychiatry 62:593–602. 10.1001/archpsyc.62.6.593 15939837 10.1001/archpsyc.62.6.593

[67] Solmi M, Radua J, Olivola M et al (2022) Age at onset of mental disorders worldwide: large-scale meta-analysis of 192 epidemiological studies. Mol Psychiatry 27:281–295. 10.1038/s41380-021-01161-7 34079068 10.1038/s41380-021-01161-7 PMC8960395

[68] Delaruelle K, Walsh SD, Dierckens M et al (2021) Mental health in adolescents with a migration background in 29 european countries: the buffering role of social capital. J Youth Adolesc 50:855–871. 10.1007/s10964-021-01423-1 33791946 10.1007/s10964-021-01423-1

[69] Helgeson VS (2003) Social support and quality of life. Qual Life Res 12 Suppl 125–31. 10.1023/A:1023509117524 10.1023/a:1023509117524 12803308

[70] Quitmann J, Rohenkohl A, Sommer R et al (2016) Explaining parent-child (dis)agreement in generic and short stature-specific health-related quality of life reports: do family and social relationships matter? Health Qual Life Outcomes 14:150. 10.1186/s12955-016-0553-0 27769269 10.1186/s12955-016-0553-0 PMC5075198

[71] Kocalevent R-D, Berg L, Beutel ME et al (2018) Social support in the general population: standardization of the Oslo social support scale (OSSS-3). BMC Psychol 6:31. 10.1186/s40359-018-0249-9 30016997 10.1186/s40359-018-0249-9 PMC6050647

[72] Buehner M (2006) Einführung in die Test- und Fragebogenkonstruktion, 2., aktualisierte und erweiterte Auflage [Introduction to test and questionnaire construction, 2nd edn. Pearson Studium, München updated and expanded ed]

[73] Johansen R, Espetvedt MN, Lyshol H et al (2021) Mental distress among young adults - gender differences in the role of social support. BMC Public Health 21:2152. 10.1186/s12889-021-12109-5 34819040 10.1186/s12889-021-12109-5 PMC8611886

[74] Bøen H, Dalgard OS, Bjertness E (2012) The importance of social support in the associations between psychological distress and somatic health problems and socio-economic factors among older adults living at home: a cross sectional study. BMC Geriatr 12:27. 10.1186/1471-2318-12-27 22682023 10.1186/1471-2318-12-27 PMC3464708

[75] Dalgard OS, Dowrick C, Lehtinen V et al (2006) Negative life events, social support and gender difference in depression: a multinational community survey with data from the ODIN study. Soc Psychiatry Psychiatr Epidemiol 41:444–451. 10.1007/s00127-006-0051-5 16572275 10.1007/s00127-006-0051-5

[76] Theron LC, Liebenberg L, Ungar M (2015) Youth resilience and culture, vol 11. Springer, Dordrecht

[77] Kim P, Evans GW, Chen E et al (2018) How socioeconomic disadvantages get under the skin and into the brain to influence health development across the lifespan. In: Halfon N, Forrest C, Lerner R, Faustman E, E. (eds) Handbook of life course health. Springer, Cham, pp 463–497 31314284

[78] Reiss F, Meyrose A-K, Otto C et al (2019) Socioeconomic status, stressful life situations and mental health problems in children and adolescents: results of the german BELLA cohort-study. PLoS ONE 14:e0213700. 10.1371/journal.pone.0213700 30865713 10.1371/journal.pone.0213700 PMC6415852

[79] Murali V, Oyebode F (2004) Poverty, social inequality and mental health. Adv psychiatr treat 10:216–224. 10.1192/apt.10.3.216

[80] Dadaczynski K, Paulus P, Nieskens B et al (2015) Gesundheit im Kontext von Bildung und Erziehung – Entwicklung, Umsetzung und Herausforderungen der schulischen Gesundheitsförderung in Deutschland [Health in an educational context: development, implementation and challenges of school health promotion in Germany]. Z f Bildungsforsch 5:197–218. 10.1007/s35834-015-0122-3

[81] Sanders MR, Kirby JN, Tellegen CL et al (2014) The triple P-Positive parenting program: a systematic review and meta-analysis of a multi-level system of parenting support. Clin Psychol Rev 34:337–357. 10.1016/j.cpr.2014.04.003 24842549 10.1016/j.cpr.2014.04.003

