# Supplementary material for: Socioeconomic Status, Protective Factors, and Mental Health Problems in Transition from Adolescence to Emerging Adulthood: Results of the Longitudinal BELLA Study
Source: Child Psychiatry Hum Dev. 2023 Aug 26;56(3):649–60. doi: 10.1007/s10578-023-01582-1 (PMC12095404; doi:10.1007/s10578-023-01582-1)
Supplement: Supplementary file 1 — Supplementary Material 1 [file 10578_2023_1582_MOESM1_ESM.pdf]

Supplementary Table 1. *Comparison of adolescents only participated at first measurement point and adolescents of the longitudinal study sample.*

	adolescents only participated at t0 (*n* = 650)			study sample: participated at t0 and t1 (*n* = 426)			X^2-tests	t-test (unpaired)
	n (%)	*M*	*SD*	*n* (%)	*M*	*SD*	Cramer's V	Cohen's d
Gender	650			426			**.119***	
male	349 (53.7)			177 (41.5)				
female	301 (46.3)			249 (58.5)				
Age in adolescence	650	14.98	1.38	426	15.17	1.28		.145$^{n.s.}$
Migration background	650			426			.045$^{n.s.}$	
migration background	43 (6.6)			19 (4.5)				
non-migration background	607 (93.4)			407 (95.5)				
SES	648	12.83	3.71	426	13.23	3.42		.112$^{n.s.}$
Self-efficacy	559	20.79	4.13	426	20.81	4.21		.005$^{n.s.}$
Family climate	554	15.87	4.62	426	15.88	4.57		.003$^{n.s.}$
Social support	536	8.42	1.69	426	8.46	1.63		.028$^{n.s.}$
MHP in adolescence	607	9.44	4.26	426	9.10	4.40		.079$^{n.s.}$
MHP in emerging adulthood (t1)	-	-	-	426	7.51	6.05	-	-

Note. t0 = first measurement point (2009-2012); t1 = 5-year follow-up (2014-2017); SES = socioeconomic status; MHP = mental health problems; n.s. = non-significant; * $p \leq .01$ (with Bonferroni-Holm correction); significant effects in bold.
